# Dynamic changes in Japan’s prevalence of abnormal findings in cervical cytology depending on birth year

**DOI:** 10.1038/s41598-018-23947-6

**Published:** 2018-04-04

**Authors:** Yutaka Ueda, Asami Yagi, Tomio Nakayama, Kei Hirai, Sayaka Ikeda, Masayuki Sekine, Etsuko Miyagi, Takayuki Enomoto

**Affiliations:** 1 Department of Obstetrics and Gynecology, Osaka University Graduate School of Medicine, 2-2 Yamadaoka, Suita, Osaka, 565-0871 Japan; 2 Department of Cancer Epidemiology, Cancer Control Center, Osaka International Cancer Institute, 3-1-69 Otemae, Chuo-ku, Osaka, 541-8567 Japan; 3 Institute for Academic Initiatives, Osaka University, Osaka, Japan, 2-2 Yamadaoka, Suita, Osaka, 565-0871 Japan; 4 Department of Gynecology, Tama-Hokubu Medical Center, Tokyo Metropolitan Health and Medical Treatment Corporation, 1-7-1 Aoba-cho, Higashimurayama, Tokyo, 189-8511 Japan; 5 Department of Obstetrics and Gynecology, Niigata University Graduate School of Medical and Dental Sciences, 1-757 Asahimachi-dori, Chuo-ku, Niigata, 951-8510 Japan; 6 Department of Obstetrics and Gynecology, Yokohama City University Graduate School of Medicine, 3-9 Fukuura, Kanazawa-ku, Yokohama, Kanagawa 236-0004 Japan

## Abstract

Japan’s governmental recommendation of HPV vaccine has now been suspended for more than 4 years. In and before 2013, the targets of 20-year-old cervical cancer screening were females born in and before 1993, i.e., those who could not have received HPV vaccination because it was not yet publicly introduced. The targets during 2014–2019 are, or will be, those born in 1994–1999, i.e., those who came of age during a period with the highest HPV immunization rate. We analyzed the statistical data for each birth year, for the cumulative HPV vaccination rates achieved as of age 16, and for the corresponding results of cervical cancer screening at age 20. The rate of abnormal findings in cervical cytology increased slightly from 3.68% in 2010 (birth year: 1990) to 4.35% in 2013 (birth year: 1993); however, it dynamically dropped to 2.99% in 2014 (birth year: 1994) and 3.03% in 2015 (birth year: 1995). In total, the rate of abnormal findings in cervical cytology was 3.96% in 2010–2013, but significantly dropped to 3.01% in 2014–2015 (*p* = 0.014). This is the first description of dynamic changes occurring in the abnormal rate of cervical cancer screening as a result of positive changes in national HPV vaccination rates.

## Introduction

The prevalence and the mortality of cervical cancer in Japan, especially for women in their twenties and thirties, are both steadily on the rise^1,2^. Japan’s public subsidy for human papilloma virus (HPV) vaccination started in 2010, permitting girls aged 13 to 16 to be immunized for only a small out-of-pocket fee. Even though it was not a school-based program, a high vaccination rate, of around 70%, was achieved across the entire country^3^. By April of 2013, HPV vaccination had nationally become a routine immunization for all girls aged 12–16.

Unfortunately, at around this same time, complaints of patients with widespread pain or movement disorders occurring after HPV immunization began to circulate, and the national news media soon began to question the vaccine’s safety. As a result, within two months the Ministry of Health, Labor and Welfare (MHLW) announced a suspension of their official recommendation for routine HPV immunization until appropriate information about HPV vaccine can be provided to the public^4^. That suspension is now more than 4 years old, despite that the World Health Organization’s (WHO’s) Global Advisory Committee on Vaccine Safety (GACVS) has stated (concerning the curcumstances in Japan) that “Policy decisions based on weak evidence, leading to lack of use of safe and effective vaccines, can result in real harm.”^5^. Subsequent to MHLW’s recommendation withdrawal, the immunization rate among girls has plummeted, and the degree of protection against HPV-associated cancers for young women now differs greatly across Japan depending on the year the female was born - relative to the initial successful public HPV vaccination program period - and to this ongoing interruption of that program.

The HPV vaccine, first publicly introduced into Japan in 2010, was not used for girls born in and before 1993, who were 17 or older at the time. During the height of the public vaccination program (2010–2013), roughly 70% of targeted eligible girls, those born between 1994 and 1999, were successfully fully immunized using public subsidy^3,6,7^. However, as a result of media-caused vaccine fears, and lack of an official recommendation, HPV vaccination has ground almost to a halt. By birth year, only 4% of girls born in 2000, who became vaccination-eligible in 2013, the year of recommendation withdrawal, have been vaccinated as of this date. But even that dismal rate has become worse, with 1% of girls born in 2001, and almost 0% of girls born after 2001, having been immunized^3,6,7^. Previously, taking into account the female’s birth year - relative to these three periods (no available vaccine, program high-vaccination, post-withdrawal low-vaccination) - we had predicted HPV-16/18 infection rates for 20-year-old girls^8^. According to those predictions, the relative risk of infection for females born from 1994 to 1999 approached 0.3.

Those girls are now older than 20, which is the standard starting age for cervical cancer screening in Japan. In Japan, all females aged 20 or older can undergo subsidized public cervical cancer screening every 2 years; those who miss their targeted year can undergo the exam the next year. In the present study, we analyzed the statistical data for each birth year, for the cumulative HPV vaccination rates, and for the corresponding results of cervical cancer screening at age 20, to evaluate effectiveness of previous introduction of HPV vaccine, which will provide strong evidence the Japanese government needs to reconsider a resumption of their recommendation for the HPV vaccine.

## Methods

In and before 2013, the targets of 20-year-old cervical cancer screening was for females born in and before 1993, i.e., those who could not have received the HPV vaccination with public subsidies because it was not yet publicly introduced. The targets during 2014–2019 are, or will be, those born in 1994–1999, i.e., those who came of age during a period with the highest HPV immunization rate (of around 70%).

In the present study, we analyzed the statistical data for each birth year, for the cumulative HPV vaccination rates achieved as of age 16, and for the corresponding results of cervical cancer screening at age 20. Data was obtained from seven local governments in Japan: Iwaki, Kawasaki, Otsu, Takatsuki, Osaka, Matsuyama, and Fukuoka. The total population of these cities is around 6–7% of that of Japan. We analyzed for annual changes in the rates of abnormal findings in cervical cytology of 20-year-old females, in association with the HPV vaccination rate of the corresponding birth year. Complete data for both 20-year-old cervical cancer screening results for 2010–2015 (birth years 1990–1995) and overall rates of HPV vaccination were available in four of the seven cities. In the present analysis, for the females who did not receive cervical cancer screening at the age of 20, the screening results from the following year, at age 21, were included. In the present study the data for cumulative vaccination and cervical cancer screening were both aggregated; however, individually, vaccination histories did not correspond to cervical cancer screening results. This is a limitation of the present study.

Fisher’s exact test was used to analyze the difference of the rates of abnormal findings in cervical cytology between the periods of 2010–2013, whose targets (birth year: 1990–1993) did not have a chance for HPV vaccination, to 2014–2015, whose targets (birth year: 1994–1995) were the generation of girls with high vaccination rates. The level of statistical significance was set at p = 0.05.

### Statements of ethics

This study was approved by the Institutional Review Board and the Ethics Committee of the Osaka University Hospital.

### Significance Statement

Japan’s governmental recommendation of the HPV vaccine has now been suspended for more than 4 years. It is timely that we evaluate the clinically relevant results from Japan’s previous introduction of the HPV vaccine. This is the first clear description of dynamic changes occurring in the abnormal rate of cervical cancer screening as a result of positive changes in national HPV vaccination rates, providing strong and credible evidence that the Japanese government needs to reconsider a resumption of their recommendation for the HPV vaccine.

## Results

**Figure 1:**
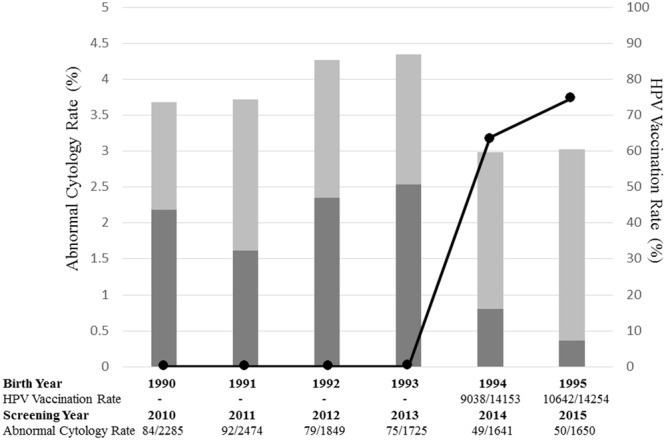
Annual change of abnormal cytology rate of cervical cancer screening at age 20, depending on HPV vaccination rate of birth year. Data for HPV vaccination and cervical cancer screening were provided from seven local governments: Iwaki, Kawasaki, Otsu, Takatsuki, Osaka, Matsuyama, and Fukuoka. Annual changes in rate of abnormal findings in cervical cytology from cervical cancer screening at the age of 20, and the cumulative HPV vaccination by the age of 16 for each birth year, were analyzed using data from Iwaki, Takatsuki, Matsuyama, and Fukuoka, which were complete for both rates for each girl born 1990 to 1995. The rate of abnormal findings in cervical cytology showing LSIL or worse could be analyzed using the data from Iwaki, Takatsuki, and Matsuyama. In Japan, all females aged 20 or older can undergo subsidized public cervical cancer screening every 2 years; those who miss their targeted year can undergo the exam the next year. In the present analysis, for the females who did not receive cervical cancer screening at the age of 20, the screening results from the following year, at age 21, were included. **Gray bar**: Annual rate of abnormal findings in cervical cytology of cervical cancer screening at the age of 20, from 2010 to 2015, with corresponding birth years of 1990 to 1995. **Dark gray bar**: Annual rate of abnormal findings in cervical cytology showing LSIL or worse of cervical cancer screening at the age of 20, from 2010 to 2015, with corresponding birth years of 1990 to 1995. **Solid line**: Cumulative initial HPV vaccination rate under public subsidies of each born year.

The rate of abnormal findings in cervical cytology increased slightly, from 3.68% in 2010 (birth year: 1990) to 4.35% in 2013 (birth year: 1993); however, the rate dropped dynamically in 2014 (birth year: 1994) to 2.99%, and held steady, 3.03%, in 2015 (birth year: 1995) in association with the public introduction of HPV vaccine (Fig. 1).

The rate of abnormal findings in cervical cytology was compared between the periods of 2010–2013, whose targets (birth year: 1990–1993) did not have a chance for HPV vaccination, to 2014–2015, whose targets (birth year: 1994–1995) were the generation of girls with high vaccination rates ranging from 63.9 to 74.7%. The rate of abnormal findings in cervical cytology (ASC-US+) was 3.96% (330/8333) in 2010–2013, but dropped to 3.01% (99/3291) in 2014–2015. This difference was statistically significant (*p* = 0.014, by Fisher’s exact test) (Table 1). The rate of LSIL+ was 2.11% (60/2841) in 2010–2013, but dropped to 0.58% (6/1032) in 2014–2015. This difference was statistically significant (p < 0.001, by Fisher’s exact test).

**Table 1:** Comparison of the rate of abnormal findings in cervical cytology and LSIL + between the generation with high vaccination rates and that with low vaccination rates.

Birth Year	1990–1993	1994–1995	p-value
Screening Year at age 20	2010–2013	2014–2015	
Rate of ASC-US +	3.96% (330/8330)	3.01% (99/3291)	0.014
Rate of LSIL+	2.11% (60/2841)	0.58% (6/1032)	<0.001

The rate of abnormal findings in cervical cytology (ASC-US+) was 3.96% (330/8333) in 2010–2013, but dropped to 3.01% (99/3291) in 2014–2015 (p = 0.014, by Fisher’s exact test). The rate of LSIL + was 2.11% (60/2841) in 2010–2013, but dropped to 0.58% (6/1032) in 2014–2015 (p < 0.001, by Fisher’s exact test).

## Discussion

Our results indicate that, as initially fully intended by the public HPV program, the yearly rate of abnormal cervical cancer screening results was statistically significantly reduced in possible correlation with the widespread administration of the HPV vaccine. However, some confounding factors have not been taken into consideration in the present analysis, i.e., the influence of teenager smoking, a potential confounding factor in many countries, seems to be limited because smoking rate of teenagers is almost zero in Japan. The protective effect of HPV vaccines against HPV-linked precancerous lesions of the cervix has now been well demonstrated around the world^9,10^. In Japan, previous studies demonstrated that the rate of abnormal findings in cervical cytology in women aged 20–24 with HPV vaccination was significantly higher than that in those without HPV vaccination^11,12^, and another study showed significant reduction of HPV-16 and 18 infection in precancerous lesions of the cervix in the vaccinated generation^13^_._ However, dynamic changes in the year over year rate of abnormal screening results in association with the introduction of the HPV vaccine, i.e., birth-year-dependent changes in cervical cancer risk, have not been previously demonstrated.

To the best of our knowledge, this is the first such clear description of the dynamic changes of the abnormal rate of cervical cancer screening as a result of positive and negative changes in HPV vaccination rates. In Japan, the standard starting age for cervical cancer screening is 20, which is among the earliest in the world, leading to this study’s earliest finding of this dynamic change. We have found more strong evidence that the government’s decision in 2010 to start public subsidies for the HPV vaccine has critically lowered the cervical cancer risk of its citizens by the present population-based study than previous vaccinated v.s. unvaccinated females studies^11,12^. Moreover, the data of cervical cancer screening exclusively at age 20 were analyzed in the present study. The results were truly pure, because young females’ sexual activities vary depending on their ages. The rate of LSIL + which are the targets for biopsy under colposcope was reduced dramatically (p < 0.001) in the generation of girls with high vaccination rates. In the present study, the data of cumulative vaccination and cervical cancer screening were aggregated ones, and did not correspond each other.

Due to alleged severe adverse events occurring after HPV immunization, MHLW announced a suspension of their official recommendation for routine HPV immunization until appropriate information about HPV vaccine can be provided to the public^4^, and the suspension is now more than 4 years old. During this period, the results of the nation-wide epidemiological study on safety of HPV vaccine funded by MHLW has already been reported that there was a certain number of unvaccinated patients with similar diverse symptoms observed after HPV immunization^14^. However, the governmental recommendation is still on suspension in Japan.

Our results, hopefully, will become the evidence the Japanese government needs to reconsider a resumption of their recommendation for the HPV vaccine. Otherwise, we foresee a looming unfortunate outcome for an entire generation of our young women. By 2019, the females born in 1994–1999 - the highly HPV vaccinated generation - will be the targets of cervical cancer screening, at the age of 20. However, in 2020, almost all females born in 2000 will reach 20 without any protective HPV vaccination, and this is/will be due entirely to the continued suspension of the governmental recommendation. If and when that recommendation is restarted, we still predict a delayed deflection in the rate of abnormal findings in cervical cytology due to current prolonged dramatically decreased HPV immunization rate following the governmental announcement of its suspension of recommendation. This cohort of unprotected women will play out as increased cases of cervical and other types of HPV-associated cancers in the coming years.

As resumption of recommendation delays for years, the relative risk of additional birth years will recapitulate the risk of the times before the vaccine was introduced^8^. Although elsewhere around the world cervical cancer is effectively being prevented with the HPV vaccine, it is still challenging here in Japan. This must change, soon.
