# Author Correction: Dynamic changes in Japan’s prevalence of abnormal findings in cervical cytology depending on birth year

**DOI:** 10.1038/s41598-018-31652-7

**Published:** 2018-09-03

**Authors:** Yutaka Ueda, Asami Yagi, Tomio Nakayama, Kei Hirai, Sayaka Ikeda, Masayuki Sekine, Etsuko Miyagi, Takayuki Enomoto

**Affiliations:** 1 Department of Obstetrics and Gynecology, Osaka University Graduate School of Medicine, 2-2 Yamadaoka, Suita, Osaka, 565-0871 Japan; 2 Department of Cancer Epidemiology, Cancer Control Center, Osaka International Cancer Institute, 3-1-69 Otemae, Chuo-ku, Osaka, 541-8567 Japan; 3 Institute for Academic Initiatives, Osaka University, Osaka, Japan, 2-2 Yamadaoka, Suita, Osaka, 565-0871 Japan; 4 Department of Gynecology, Tama-Hokubu Medical Center, Tokyo Metropolitan Health and Medical Treatment Corporation, 1-7-1 Aoba-cho, Higashimurayama, Tokyo, 189-8511 Japan; 5 Department of Obstetrics and Gynecology, Niigata University Graduate School of Medical and Dental Sciences, 1-757 Asahimachi-dori, Chuo-ku, Niigata, 951-8510 Japan; 6 Department of Obstetrics and Gynecology, Yokohama City University Graduate School of Medicine, 3-9 Fukuura, Kanazawa-ku, Yokohama, Kanagawa 236-0004 Japan

Correction to: *Scientific Reports* 10.1038/s41598-018-23947-6, published online 04 April 2018

The original version of this Article contained an error in the title of the paper, where the word “cervical” was duplicated. This has now been corrected in the PDF and HTML versions of the Article.

